# Supplementary material for: ‘I Was Present but I Was Absent’: Perceptions and Experiences of the Non-Medical Use of Prescription or over the Counter Medication among Employed South African Women
Source: Int J Environ Res Public Health. 2022 Jun 10;19(12):7151. doi: 10.3390/ijerph19127151 (PMC9222615; doi:10.3390/ijerph19127151)
Supplement: Supplementary file 1 [file ijerph-19-07151-s001.zip › ijerph-1757724-supplementary.pdf]

ANNEXURE B:
INTERVIEW GUIDE
Semi-structured interviews with women who have a history of NMIU/OTCPRES use.

Objective 3:

For objective 3, we aim to interview employed women who have a history of NMIU/OTCPRES use and explore possible barriers they experience or have experienced in accessing help for NMIU/OTCPRES use and determine perceived facilitators for accessing help.

Demographic Information

Age:	
Work Area:	
Are you in a management/supervisory position? Yes/No	
How long have your worked for? No of years:	
Are you married/single/have a partner?	
Do you have children?	

1. What is your experience of using over-the-counter prescription medication for non-medically indicated use? *(note: probe into, first use, why they strated using, when and if they ovever stopped an why, reasons for use, why the sought assistance and the role of the workplace in providing assistance. Probe into, type of medication, frequency of use, mode of use)*
    a. Why were you initially prescribed these medications?
    b. Why do you continue to these medications?
    c. How does it help you in your day-to-day life?
    d. How have you coped in the past without these medications?
2. What kind of problems have you experienced as a result of using these medications?
    a. In which ways are these medications helping you to cope?
        i. Physically
        ii. Mentally?
        iii. Psychologically?
        iv. Socially?
        v. Emotionally?
3. Who initially referred you for help?
4. How would you describe your relationship with your prescribing doctor and pharmacist?
5. Did you feel confident in discussing issues pertaining to your reliance on the medications with your doctor or pharmacist?

6. Do you think it would be important for someone to discuss possible contra-indications of medicines with their doctor or pharmacist and why?
7. How did taking these medications benefit you with regards to your professional/career/family/personal life?
8. Did you know anything about the dangers of meds prior to seeking treatment…what did you know?
9. How, in your opinion, readily available is information about the dangers about non-medically indicated use of over-the-counter prescription medication? Do you think more can be done?
10. What was your experience with access to treatment, was it easy to obtain treatment? If not:
    a. What barriers have you encountered that hindered your access, admission or procurement of treatment services?
    b. What were possible enablers to seeking treatment?
11. We are creating more awareness about non-medically indicated use of over-the-counter prescription medications. In doing so, we would like to take advantage of technology using applications and websites to promote awareness.

    What are your thoughts about using technology, and web-based applications and websites to promote awareness about the dangers of using over-the-counter prescription medication for non-medical reasons?
    a. How do you think using technology will help with health promotion and awareness in this regard? *(Note: probe towards stigma, and whether having a web-based versus a programme would help buffer this).*
    b. Is there anything else you would like to share with us?
